# Floral Response to Heat: A Study of Color and Biochemical Adaptations in Purple Chrysanthemums

**DOI:** 10.3390/plants13131865

**Published:** 2024-07-05

**Authors:** Fenglan Wang, Zhimei Li, Qing Wu, Yanhong Guo, Jun Wang, Honghui Luo, Yiwei Zhou

**Affiliations:** 1 College of Horticulture and Landscape Architecture, Zhongkai Agricultural Engineering College, Guangzhou 510408, China; wangfl2002@163.com (F.W.); lizm202407@163.com (Z.L.);; 2 Guangdong Key Lab of Ornamental Plant Germplasm Innovation and Utilization, Environmental Horticulture Research Institute, Guangdong Academy of Agricultural Sciences, Guangzhou 510640, China

**Keywords:** chrysanthemum, high temperature, flower color, anthocyanin, biosynthesis, degradation

## Abstract

Chrysanthemums are among the world’s most popular cut flowers, with their color being a key ornamental feature. The formation of these colors can be influenced by high temperatures. However, the regulatory mechanisms that control the fading of chrysanthemum flower color under high-temperature stress remain unclear. This study investigates the impact of high temperatures on the color and biochemical responses of purple chrysanthemums. Four purple chrysanthemum varieties were exposed to both normal and elevated temperature conditions. High-temperature stress elicited distinct responses among the purple chrysanthemum varieties. ‘Zi Feng Che’ and ‘Chrystal Regal’ maintained color stability, whereas ‘Zi Hong Tuo Gui’ and ‘Zi lian’ exhibited significant color fading, particularly during early bloom stages. This fading was associated with decreased enzymatic activities, specifically of chalcone isomerase (CHI), dihydroflavonol 4-reductase (DFR), and anthocyanidin synthase (ANS), indicating a critical period of color development under heat stress. Additionally, the color fading of ‘Zi Lian’ was closely related to the increased activity of the peroxidase (POD) and polyphenol oxidase (PPO). Conversely, a reduction in β-glucosidase (βG) activity may contribute significantly to the color steadfastness of ‘Zi Feng Che’. The genes Cse_sc027584.1_g010.1 (*PPO*) and Cse_sc031727.1_g010.1 (*POD*) might contribute to the degradation of anthocyanins in the petals of ‘Zi Hong Tuo Gui’ and ‘Zi Lian’ under high-temperature conditions, while simultaneously maintaining the stability of anthocyanins in ‘Zi Feng Che’ and ‘Chrystal Regal’ at the early bloom floral stage. The findings of this research provide new insights into the physiological and biochemical mechanisms by which chrysanthemum flower color responds to high-temperature stress.

## 1. Introduction

The chrysanthemum (*Chrysanthemum* × *morifolium*), a perennial herbaceous plant of the genus *Chrysanthemum* in the Asteraceae family, holds the second position in the global floriculture industry [1,2]. It is renowned for its vibrant color palette, which includes white, yellow, pink, orange, red, and purple–red [3,4,5,6,7]. Anthocyanins, the key pigments, influence the color from red to purple–red [1,8,9].

The biosynthetic pathway and key genes of anthocyanins in plants have been widely studied. The synthesis of anthocyanins involves three stages. Initially, the precursor phenylalanine is catalyzed by phenylalanine ammonia-lyase (PAL) to produce 4-coumaroyl-CoA and malonyl-CoA. The second stage involves the formation of chalcones through the catalysis by chalcone synthase (CHS) and chalcone isomerase (CHI). These chalcones are further catalyzed by flavanone 3-hydroxylase (F3H), flavonoid 3′-hydroxylase (F3′H), and flavonoid-3′,5′-hydroxylase (F3′5′H) to produce dihydrokaempferol, dihydroquercetin, and dihydromyricetin. In the final stage, these substances are reduced by dihydroflavonol 4-reductase (DFR) to colorless anthocyanins, which are then catalyzed by anthocyanidin synthase (ANS) to form colored floral pigments [10,11,12].

Extracellular anthocyanin degradation is predominantly influenced by light and heat. Studies on photodegradation pathways indicate that the final products of light-induced anthocyanin degradation are the same as those resulting from thermal degradation, although the pathways themselves differ and remain to be fully elucidated [13,14,15]. Research has identified key enzymes involved in anthocyanin degradation, including polyphenol oxidase (PPO), peroxidase (POD), β-glucosidase (βG), and pectinase, which likely orchestrate intracellular anthocyanin degradation [16,17,18]. For instance, Zhang et al. [19] demonstrated that the βG enzyme in litchi pericarp facilitates the cleavage of the βG bond in anthocyanins, potentially contributing to pericarp browning. Hou et al. [20] observed that the degradation rates of three cyanidin glycosides and one peonidin glycoside in black rice increase with rising environmental temperatures. Similarly, in *Brunfelsia calycina*, POD enzyme activity correlates with the progressive degradation of anthocyanins [21].

High temperatures also impinge upon anthocyanins’ synthesis in chrysanthemum petals. Nozaki et al. [22] explored the effects of high temperatures on flower color and anthocyanin levels of six pink chrysanthemum varieties, finding that they exhibited diminished concentrations of cyanidin 3-*O*-(6″-*O*-monomalonyl-β-glucopyranoside) and cyanidin 3-*O*-(3″,6″-*O*-dimalonyl-β-glucopyranoside) at elevated temperatures, resulting in a notably paler petal color at full bloom at 30 °C compared to 20 °C. Moreover, when chrysanthemums grow at temperatures above 30 °C, their petal anthocyanin content significantly decreases, although different varieties respond differently to temperature. For instance, ‘Sei-Monako’ and ‘Sei-Suffle’ experienced a marked decrease in anthocyanin content following identical high-temperature treatments, whereas ‘Chatoo’ was less affected [22]. Sun et al. [23] identified six *WRKY* genes in the transcriptome of heat-treated chrysanthemum leaves that are transcriptionally responsive to high-temperature stress, suggesting that CnWRKYs and CnMYB may play roles in modulating HSF or HSP signal transduction. Further investigation revealed that CmMYB012 in chrysanthemums downregulates the expression of *CmCHS*, *CmDFR*, *CmANS*, and *CmUFGT* under high temperatures, leading to decreased anthocyanin synthesis and a lighter flower color [24]. Nonetheless, the regulatory mechanisms governing chrysanthemum flower color fading under high-temperature stress, encompassing both anthocyanin degradation and biosynthesis, are not yet fully understood.

The high-temperature-induced fading of chrysanthemum petals during cultivation significantly compromises the quality of chrysanthemum products. This study examines four representative purple chrysanthemum varieties, with anthocyanins as the primary pigment, including two varieties susceptible to fading under high temperatures, ‘Zi Hong Tuo Gui’ and ‘Zi Lian’, and two varieties resistant to fading, ‘Zi Feng Che’ and ‘Chrystal Regal’. Under standard (day 24 °C/night 15 °C) and elevated (day 35 °C/night 26 °C) temperature conditions, the study assesses changes in flower color, anthocyanin content, and the activity of enzymes associated with anthocyanin biosynthesis and degradation across various flower development stages (bud, S1; early bloom, S2; early full bloom, S3; mid-full bloom, S4). Furthermore, we conduct quantitative real-time PCR (qRT-PCR) analyses to investigate the expression changes of key genes involved in the synthesis and degradation of anthocyanins. These analyses are crucial for elucidating the mechanisms responsible for the discoloration of chrysanthemum petals subjected to high-temperature conditions.

## 2. Results

### 2.1. Changes in Chrysanthemum Flower Color Phenotypes after High-Temperature Treatments

**Figure 1:**
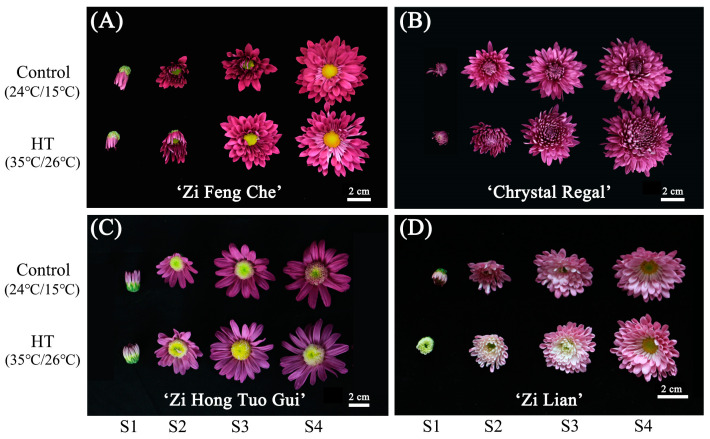
Phenotypic changes in four purple chrysanthemum varieties subjected to high-temperature treatment. Panels (**A**–**D**) represent ‘Zi Feng Che’, ‘Crystal Regal’, ‘Zi Hong Tuo Gui’, and ‘Zi Lian’, respectively.

Initially, we examined the phenotypic alterations in flower color among four purple chrysanthemum varieties subjected to high-temperature stress. After seven days of exposure to high (35 °C/26 °C) and normal (24 °C/15 °C) temperatures, the phenotypic changes in chrysanthemum flower color were as depicted in Figure 1. In comparison to the control group, the high-temperature treatment (HT) did not significantly alter the flower color phenotype of ‘Zi Feng Che’ (Figure 1A) across all stages (S1, S2, S3, and S4). ‘Chrystal Regal’ (Figure 1B) showed minimal fading, although petal curling was observed. ‘Zi Hong Tuo Gui’ (Figure 1C) exhibited fading across all stages, and ‘Zi Lian’ (Figure 1D) transitioned from pink–purple to pale pink or white, with stage S1 petals almost entirely white, stage S3 outer petals pink, and inner petals white. However, stage S4 petals remained unchanged compared to the control. Notably, ‘Zi Hong Tuo Gui’ (Figure 1C) developed stage S4 from the normally colored stage S2 flowers, with petals noticeably shifting from purple to pink and becoming distorted, and the color was significantly lighter than the control’s stage S4. ‘Zi Lian’ (Figure 1D) developed stage S3 from normally colored stage S1 flowers, with new inner petals appearing significantly lighter than the control’s stage S3 inner petals. The high-temperature-sensitive varieties, ‘Zi Hong Tuo Gui’ and ‘Zi Lian’, experienced petal fading within seven days of high-temperature stress, markedly diminishing their ornamental appeal.

### 2.2. Changes in Petal Total Anthocyanin, Flavonoid, and Phenolic Content after High-Temperature Treatment

**Figure 2:**
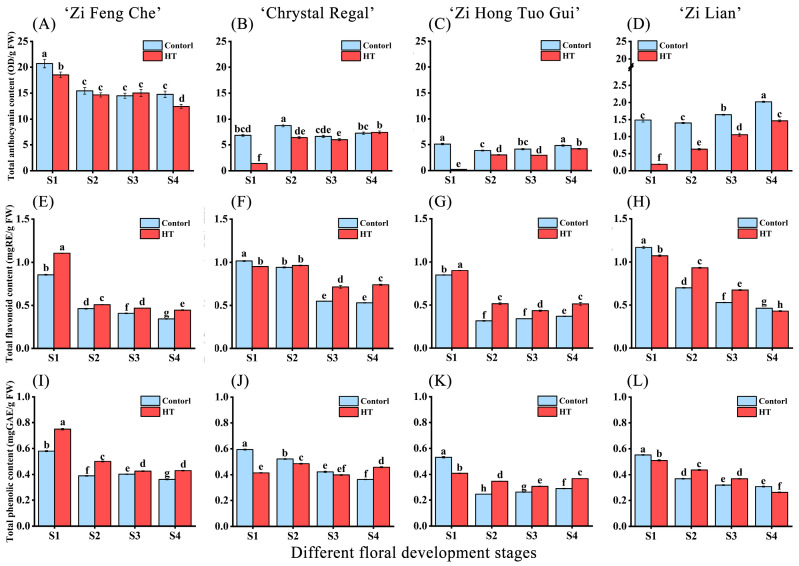
Changes in total anthocyanin content (TAC), total flavonoid content (TFC), and total phenolic content (TPC) following high-temperature treatment during different floral development stages of four purple chrysanthemum varieties. (**A**,**E**,**I**) Variations in TAC, TFC, and TPC for ‘Zi Feng Che’. (**B**,**F**,**J**) Variations in TAC, TFC, and TPC for ‘Chrystal Regal’. (**C**,**G**,**K**) Variations in TAC, TFC, and TPC for ‘Zi Hong Tuo Gui’. (**D**,**H**,**L**) Variations in TAC, TFC, and TPC for ‘Zi Lian’. Differences denoted by distinct lowercase letters are statistically significant at the *p* < 0.05 level.

To elucidate the color changes of four purple chrysanthemum varieties under high-temperature stress, we employed spectrophotometry to measure the total anthocyanin content in the petals. The determination of anthocyanin content (Figure 2) revealed significant differences among the varieties, with the overall content decreasing in the following order: ‘Zi Feng Che’ > ‘Chrystal Regal’ > ‘Zi Hong Tuo Gui’ > ‘Zi Lian’. Compared to the control, the relative anthocyanin content (TAC) of chrysanthemum flowers at each developmental stage decreased to varying degrees under high-temperature stress, with stage S1 being the most affected.

Under high-temperature stress, ‘Zi Feng Che’ (Figure 2A) exhibited a TAC decrease at stages S1 and S4, with no significant change at stages S2 and S3, maintaining relatively high levels throughout. The highest TAC in the control was observed in stage S1 petals, at 20.68 OD·g^−1^ (FW), and the lowest in the high-temperature group’s stage S4 petals, at 12.44 OD·g^−1^ (FW). ‘Chrystal Regal’ (Figure 2B) experienced a significant TAC decrease at stages S1 and S2, with reductions of 79.23% and 26.65%, respectively, compared to the control. Stages S3 and S4 showed no significant decrease. ‘Zi Hong Tuo Gui’ (Figure 2C) displayed a significant TAC decrease across all stages, with the most substantial reduction in stage S1 (95.66% compared to the control). ‘Zi Lian’ generally had a lower TAC, with the highest content in the control S4 stage (2.02 OD·g^−1^ FW; Figure 2D). All stages experienced a significant decrease under high-temperature stress, particularly stage S1, which showed an 87.20% reduction compared to the control stage. In summary, ‘Zi Feng Che’ was least affected by high-temperature stress, while the other three varieties exhibited significant effects on TAC from stage S1 to full bloom (S3), especially the high-temperature-sensitive ‘Zi Hong Tuo Gui’ and ‘Zi Lian’.

Additionally, we measured the total flavonoid content (TFC) and total phenolic content (TPC) of the petals. The results indicated that ‘Zi Feng Che’ experienced a slight increase in TFC (Figure 2E) and TPC (Figure 2I) from the bud stage (S1) to full bloom (S4) under high-temperature stress, with consistent trends. The most significant impact on TFC and TPC was during the bud stage (S1), with increases of 0.25 mgRE/g and 0.17 mgGAE/g, respectively, in the high-temperature group compared to the control. ‘Chrystal Regal’ showed a slight decrease in TFC (Figure 2F) and TPC (Figure 2J) during the bud stage under high-temperature treatment, but a slight increase during the full-bloom stage (S4). ‘Zi Hong Tuo Gui’ displayed a slight increase in TFC (Figure 2G) across all stages in the high-temperature group compared to the control, but its TPC (Figure 2K) showed an opposite trend at stage S1, with similar trends to the TFC in the other stages. ‘Zi Lian’ exhibited consistent trends in TFC (Figure 2H) and TPC (Figure 2L) changes across all developmental stages between the high-temperature group and the control, with slightly lower content in stages S1 and S4, but slightly higher at stages S2 and S3 in the high-temperature group. Overall, high-temperature stress did not exhibit a clear pattern of impact on the TFC and TPC during each developmental stage of the four chrysanthemum varieties, with only minor content differences, suggesting that TFC and TPC changes may not be pivotal in the petal discoloration process under high temperatures.

### 2.3. Changes in Enzyme Activity Related to Anthocyanin Biosynthesis after High-Temperature Treatment

**Figure 3:**
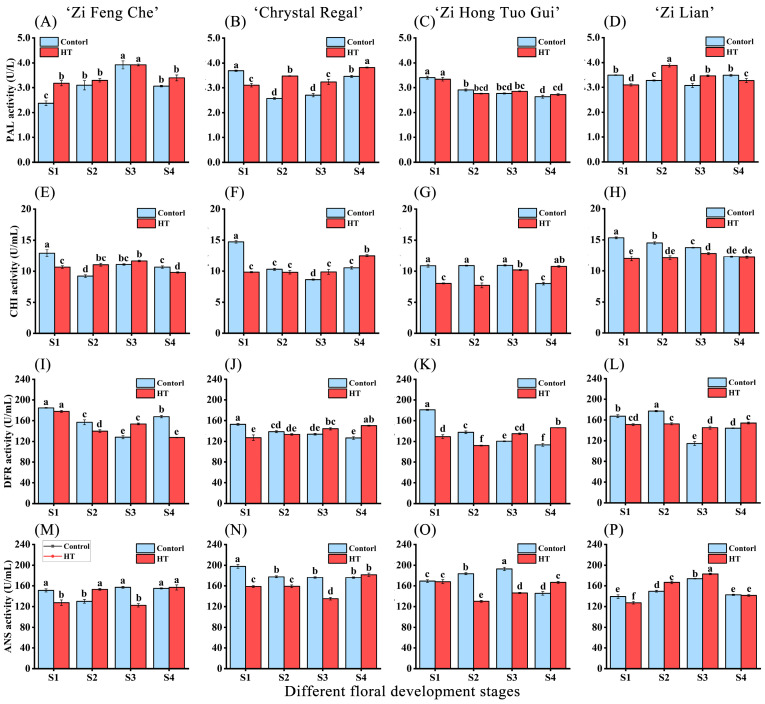
Changes in phenylalanine ammonia-lyase (PAL), chalcone isomerase (CHI), dihydroflavonol 4-reductase (DFR), and anthocyanidin synthase (ANS) following high-temperature treatment during different floral development stages of four purple chrysanthemum varieties. (**A**,**E**,**I**,**M**) Variations in PAL, CHI, DFR, and ANS for ‘Zi Feng Che’. (**B**,**F**,**J**,**N**) Variations in PAL, CHI, DFR, and ANS for ‘Chrystal Regal’. (**C**,**G**,**K**,**O**) Variations in PAL, CHI, DFR, and ANS for ‘Zi Hong Tuo Gui’. (**D**,**H**,**L**,**P**) Variations in PAL, CHI, DFR, and ANS for ‘Zi Lian’. Differences denoted by distinct lowercase letters are statistically significant at the *p* < 0.05 level.

PAL, CHI, DFR, and ANS are key enzymes in the biosynthetic pathway related to plant anthocyanin synthesis. To further identify which enzymes were affected by high-temperature stress, leading to changes in anthocyanin content, we assessed the enzymatic activities of PAL, CHI, DFR, and ANS. In varieties resistant to high-temperature-induced fading, ‘Zi Feng Che’ showed no significant reduction in PAL enzyme activity across all petal developmental stages after high-temperature treatment compared to the control. CHI enzyme activity experienced a marked decrease during the S1 and S4 stages, most notably in the S1 stage, with a reduction of 17.45% compared to the control S1 stage (Figure 3A,E). DFR enzyme activity was significantly reduced during the S1, S2, and S4 stages, with the most pronounced decrease of 23.93% in the S4 stage compared to the control S4 stage. ANS enzyme activity was significantly lowered by 15.63% in the S1 stage and by 22.03% in the S3 stage (Figure 3I,M). Conversely, ‘Chrystal Regal’ exhibited a significant decline in PAL and CHI enzyme activities solely in the S1 stage following high-temperature treatment, with CHI enzyme activity demonstrating a more significant decrease of 33.26% (Figure 3B,F). DFR enzyme activity was significantly reduced by 16.81% in the S1 stage and by 3.81% in the S2 stage. ANS enzyme activity was significantly diminished during the S1, S2, and S3 stages, with more considerable reductions of 19.78% and 23.32% in the S1 and S3 stages, respectively (Figure 3J,N).

In varieties susceptible to high-temperature-induced fading, ‘Zi Hong Tuo Gui’ exhibited no significant difference in PAL enzyme activity across all stages after high-temperature treatment compared to the control. Nonetheless, CHI enzyme activity markedly decreased during the S1, S2, and S3 stages, with the most pronounced reductions of 26.11% and 29.08% in the S1 and S2 stages, respectively (Figure 3C,G). DFR enzyme activity substantially declined in the S1 and S2 stages, with the most notable decrease of 28.56% in the S1 stage relative to normal-temperature conditions. ANS enzyme activity significantly fell by 29.11% in the S2 stage and by 24.25% in the S3 stage (Figure 3K,O). ‘Zi Lian’ demonstrated a significant decrease in PAL enzyme activity solely in the S1 stage following high-temperature treatment. CHI enzyme activity significantly diminished during the S1, S2, and S3 stages, with the greatest decrease of 17.45% in the S1 stage (Figure 2D,H). DFR enzyme activity notably decreased by 9.71% in the S1 stage and by 13.88% in the S2 stage. ANS enzyme activity significantly dropped by 8.58% in the S1 stage (Figure 3L,P). Collectively, high-temperature stress differentially impacted the enzyme activities of PAL, CHI, DFR, and ANS involved in anthocyanin synthesis in chrysanthemum petals across varieties and developmental stages. The influence was more substantial on CHI, DFR, and ANS enzyme activities, especially during the S1 and S2 stages, indicating these stages as potential critical junctures for enzyme activity influencing anthocyanin biosynthesis transformations.

### 2.4. Changes in Enzyme Activities Related to Anthocyanin Degradation after High-Temperature Treatment

**Figure 4:**
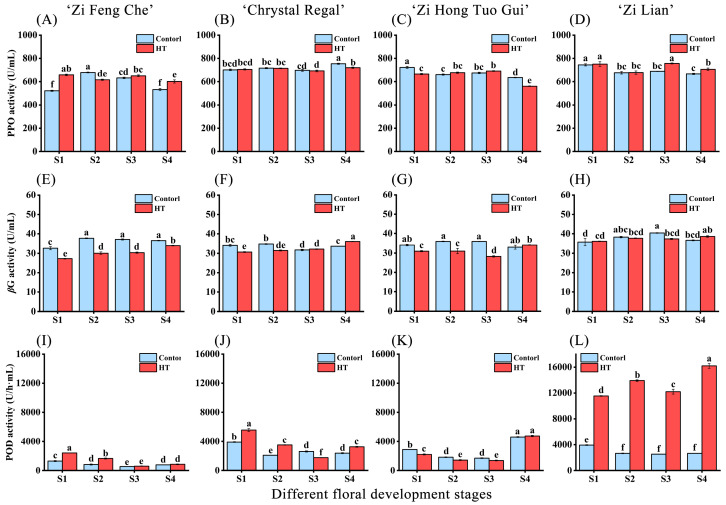
Changes in polyphenol oxidase (PPO), β-glucosidase (βG), and peroxidase (POD) enzyme activities following high-temperature treatment during different floral development stages of four purple chrysanthemum varieties. (**A**,**E**,**I**) Variations in PPO, βG, and POD enzyme activities for ‘Zi Feng Che’. (**B**,**F**,**J**) Variations in PPO, βG, and POD enzyme activities for ‘Chrystal Regal’. (**C**,**G**,**K**) Variations in PPO, βG, and POD enzyme activities for ‘Zi Hong Tuo Gui’. (**D**,**H**,**L**) Variations in PPO, βG, and POD enzyme activities for ‘Zi Lian’. Differences denoted by distinct lowercase letters are statistically significant at the *p* < 0.05 level.

Key enzymes in the anthocyanin synthesis pathway directly influence anthocyanin accumulation, while PPO, POD, and βG enzymes may contribute to anthocyanin degradation. To discern whether these degradative enzymes in chrysanthemums were altered under high-temperature stress, we also measured the activities of these three enzymes. Compared to the control, ‘Zi Feng Che’ exhibited a significant increase in PPO enzyme activity during the S1, S3, and S4 stages after high-temperature treatment, especially in the S1 stage, with an increase of 26.32% compared to normal-temperature conditions. However, βG enzyme activity significantly waned across all stages, suggesting that the overall stability of anthocyanin accumulation in this variety’s petals might be correlated with the diminished activity of degradation-associated enzymes (Figure 4A,E). ‘Chrystal Regal’ displayed relatively consistent PPO and βG enzyme activities across all stages following high-temperature stress, without discernible trends (Figure 4B,F). In varieties prone to high-temperature-induced fading, ‘Zi Hong Tuo Gui’ experienced a significant decline in PPO enzyme activity during the S1 and S4 stages and a significant reduction in βG enzyme activity during the S1–S3 stages after high-temperature stress (Figure 4C,G). ‘Zi Lian’ encountered a significant surge in PPO enzyme activity during the S3 and S4 stages, with respective increases of 9.83% and 5.77% compared to the control. βG enzyme activity remained relatively stable across all stages, implying that βG enzyme might be implicated in the high-temperature-induced fading phenomenon in ‘Zi Hong Tuo Gui’ but not directly linked with ‘Zi Lian’ (Figure 4D,H).

Regarding POD enzyme activity, ‘Zi Feng Che’ registered a significant boost during the S1 and S2 stages after high-temperature stress, with respective increments of 84.47% and 95.53% compared to the control (Figure 4I). ‘Chrystal Regal’ underwent a significant rise in POD enzyme activity during the S1, S2, and S4 stages after high-temperature stress, with respective increases of 42.95%, 67.33%, and 36.17% (Figure 4J). ‘Zi Hong Tuo Gui’ displayed no significant alteration in POD enzyme activity under high-temperature stress compared to normal-temperature conditions, yet both temperature treatments led to a significant escalation in POD enzyme activity during the S4 stage, with the high-temperature group exhibiting a 242.94% increase compared to the S3 stage (Figure 4K). ‘Zi Lian’ showed a significant upsurge in POD enzyme activity across all stages following high-temperature stress, with respective increases of 193.24%, 420.58%, 380.03%, and 507.05% compared to the control (Figure 4L). In summation, ‘Zi Feng Che’ maintained the lowest POD enzyme activity across all stages, whereas ‘Zi Lian’ manifested significantly heightened POD enzyme activity after high-temperature stress in comparison to the other three varieties.

### 2.5. Indentification of Important Indicators with Significant Changes after High-Temperature Treatment

**Figure 5:**
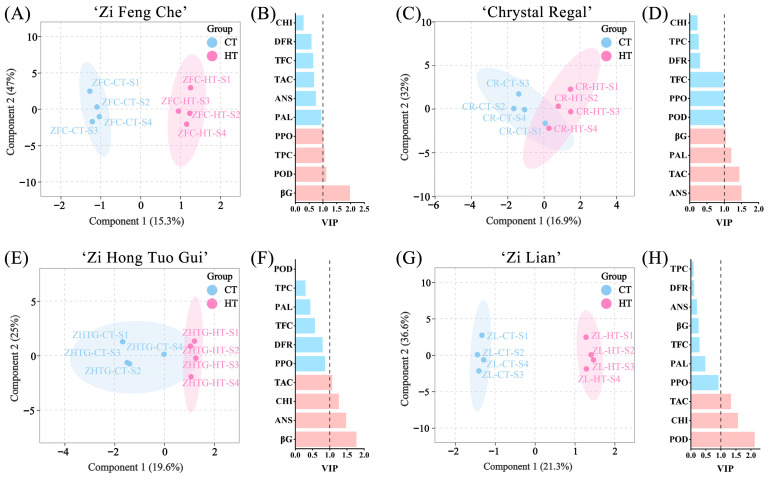
Orthogonal Partial Least Squares Discriminant Analysis (OPLS-DA) between control and high-temperature treatment for four purple chrysanthemum varieties. (**A**,**C**,**E**,**G**) OPLS-DA score plot for ‘Zi Feng Che’, ‘Chrystal Regal’, ‘Zi Hong Tuo Gui’, and ‘Zi Lian’, respectively. (**B**,**D**,**F**,**H**) Variable Importance in Projection (VIP) values for 10 physiological indicators for ‘Zi Feng Che’, ‘Chrystal Regal’, ‘Zi Hong Tuo Gui’, and ‘Zi Lian’, respectively.

To discern physiological indicators that underwent significant changes after high-temperature treatment, an Orthogonal Partial Least Squares Discriminant Analysis (OPLS-DA) was performed. The OPLS-DA results, as depicted in Figure 5, revealed substantial alterations in ten physiological indicators of chrysanthemum petals under high-temperature treatment across four cultivars. In ‘Zi Feng Che’, ‘Zi Hong Tuo Gui’, and ‘Zi Lian’, the score plots distinctly separated the high-temperature and normal-temperature treatments. Conversely, ‘Chrystal Regal’ exhibited overlapping confidence ellipses between HT and CT in its score plot. VIP results indicated that in ‘Zi Feng Che’, four variables with VIP values exceeding 1 included BGA, POD, TPC, and PPO. For ‘Chrystal Regal’, four variables with VIP values exceeding 1 were ANS, TAC, PAL, and BGA. ‘Zi Hong Tuo Gui’ had BGA, ANS, CHI, and TAC, while ‘Zi Lian’ had POD, CHI, and TAC. These VIP results affirm the significance of these indicators with elevated VIP values in response to high temperatures.

### 2.6. Correlation Analysis between Different Physiological Indicators

**Figure 6:**
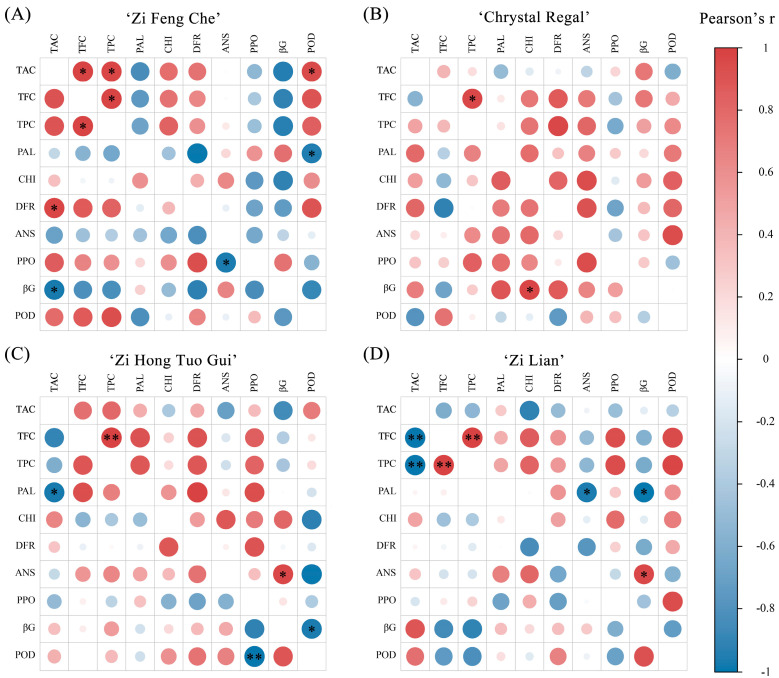
Pearson correlation analysis of ten physiological indicators for four purple chrysanthemum varieties under normal- and high-temperature conditions. (**A**–**D**) Correlation analysis of physiological indicators for ‘Zi Feng Che’, ‘Chrystal Regal’, ‘Zi Hong Tuo Gui’, and ‘Zi Lian’, respectively. The upper triangle indicates correlation coefficients under normal-temperature conditions, while the lower triangle shows the coefficients after high-temperature treatment. Significance levels are denoted as follows: “*” for *p* < 0.05 and “**” for *p* < 0.01.

To clarify the relationship between TAC, TFC, TPC, and the activities of enzymes associated with anthocyanin synthesis and degradation, a Pearson correlation analysis was conducted on the variations in TAC, TFC, TPC, and key enzyme activities for each cultivar (Figure 6; Tables S1–S4). In ‘Zi Feng Che’, under normal-temperature conditions, TAC, TFC, and TPC demonstrated significant positive intercorrelations (Figure 6A; Table S1). TAC showed positive correlations with CHI, DFR, and ANS enzyme activities and negative correlations with PPO and βG enzymes. Under high-temperature conditions, TFC and TPC maintained significant positive correlations, and TAC was positively correlated with CHI enzyme activity and significantly with DFR enzyme activity, while exhibiting significant negative correlations with βG enzyme and between PPO enzyme and ANS enzyme activities. In ‘Chrystal Regal’, TFC and TPC were significantly positively correlated under normal-temperature conditions; under high-temperature conditions, TAC correlated positively with PAL, CHI, DFR, and ANS enzymes involved in anthocyanin synthesis and negatively with POD enzyme (Figure 6B; Table S2). In ‘Zi Hong Tuo Gui’, TFC and TPC were significantly positively correlated under normal-temperature conditions, and TAC correlated positively with PAL and DFR enzyme activities and negatively with βG enzyme (Figure 6C; Table S3). Under high-temperature conditions, TAC correlated positively with CHI and DFR enzyme activities and negatively with PPO enzyme. In ‘Zi Lian’, under control conditions, TAC correlated positively with PAL enzyme activity and negatively with PPO, POD, and βG enzymes, with βG enzyme showing a significant negative correlation with PAL and ANS (Figure 6D; Table S4). In the high-temperature group, anthocyanins correlated positively with PAL, CHI, DFR, and ANS enzymes related to anthocyanin synthesis, and negatively with PPO enzyme.

### 2.7. Genes Expression Analysis at Early Full-Bloom Stage

**Figure 7:**
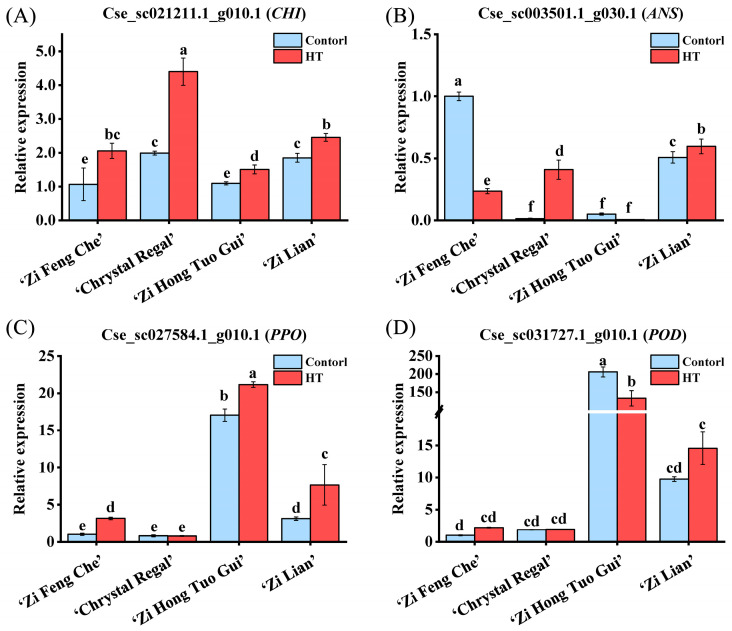
qRT-PCR analysis of four key genes for four purple chrysanthemum varieties under normal- and high-temperature conditions at the early bloom floral stage. (**A**–**D**) Expression analysis of Cse_sc021211.1_g010.1 (*CHI*), Cse_sc003501.1_g030.1 (*ANS*), Cse_sc027584.1_g010.1 (*PPO*), and Cse_sc031727.1_g010.1 (*POD*) genes. Differences denoted by distinct lowercase letters are statistically significant at the *p* < 0.05 level.

To elucidate the gene expression patterns pertinent to anthocyanin biosynthesis and catabolism after high-temperature stress, we conducted an expression analysis of *CHI*, *ANS*, *PPO*, and *POD* genes during the S3 stage across four chrysanthemum cultivars, as depicted in Figure 7. After high-temperature exposure, a consistent upregulation of the Cse_sc021211.1_g010.1 (*CHI*) gene was observed across all cultivars. The Cse_sc003501.1_g030.1 (*ANS*) gene exhibited an upregulation in ‘Chrystal Regal’ and ‘Zi Lian‘, contrasting with a downregulation in ‘Zi Feng Che’ and ‘Zi Hong Tuo Gui’. The Cse_sc027584.1_g010.1 (*PPO*) gene was upregulated in ‘Zi Feng Che’, ‘Zi Hong Tuo Gui’, and ‘Zi Lian’, with ‘Chrystal Regal’ showing no notable change. For the Cse_sc031727.1_g010.1 (*POD*) gene, ‘Zi Feng Che’ and ‘Zi Lian’ demonstrated no significant expression alterations, whereas it was downregulated in ‘Zi Hong Tuo Gui’ and upregulated in ‘Zi Lian’. These findings imply that the *PPO* and *POD* genes may facilitate the degradation of anthocyanins in the petals of ‘Zi Hong Tuo Gui’ and ‘Zi Lian’ under elevated temperatures, while preserving anthocyanin stability in ‘Zi Feng Che’ and ‘Chrystal Regal’.

## 3. Discussion

High temperatures are known to influence anthocyanin metabolism. For example, elevated temperatures have been observed to reduce anthocyanin concentrations in apple fruits [25] and to diminish anthocyanin accumulation in grape berry skins during nighttime [26]. This is primarily attributed to the suppression of genes encoding enzymes in the flavonoid biosynthesis pathway by high temperatures, a phenomenon that has been the subject of numerous studies aiming to understand the underlying mechanisms. Our research corroborates these findings, indicating that high-temperature stress results in reduced anthocyanin content in chrysanthemum petals.

In our investigation, the high-temperature-sensitive chrysanthemum ‘Zi Hong Tuo Gui’ underwent a color transition from purple to pale pink from stages S1 to S4 under high-temperature stress (35 °C/26 °C), accompanied by a decrease in anthocyanin content. Similarly, ‘Zi Lian’ exhibited a color change from pink to white in the inner petals from stages S1 to S3, with a notable reduction in anthocyanin content. However, no significant difference in petal coloration and anthocyanin content was observed at stage S4 between high-temperature and normal-temperature conditions. This suggests that chrysanthemums may experience petal fading within seven days of high-temperature stress, likely due to decreased anthocyanin synthesis and increased degradation, echoing the findings of Nozaki et al. [22], where heat treatment at 30 °C resulted in reduced cyanidin-3-*O*-glucosides and subsequent petal fading during the full bloom. In contrast, the high-temperature-stable chrysanthemum ‘Zi Feng Che’ retained relatively high anthocyanin levels despite a slight decrease after high-temperature stress, with no significant change in petal color. ‘Chrystal Regal’ showed a considerable decrease in anthocyanin content during stages S1 and S2 after high-temperature stress, yet the phenotypic appearance altered minimally, displaying only a slight lightening of the flower color. These observations indicate that chrysanthemum varieties exhibit varying degrees of sensitivity to high temperatures, with high-temperature-sensitive varieties experiencing a significant reduction in anthocyanin content under stress, while high-temperature-stable varieties are less affected, aligning with the findings of Ohmiya [7].

Temperature is a critical factor in anthocyanin stability, influencing both the synthesis process and the stability of anthocyanins. High temperatures hinder the activity of enzymes involved in anthocyanin synthesis, reducing the rate of synthesis, and enhance anthocyanin degradation, leading to a significant decrease in cellular anthocyanin accumulation [27]. Our study’s results are in agreement, showing that high temperatures affect enzymes related to both anthocyanin synthesis and degradation. We observed that the enzyme activities of PAL, CHI, DFR, and ANS in the petals of the four chrysanthemum varieties displayed varying responses under high and normal temperatures, with the most pronounced effect on CHI, DFR, and ANS enzyme activities under high-temperature stress. Notably, CHI enzyme activity significantly decreased in the S1 stage across all varieties under high temperatures compared to the control, suggesting an inhibition of the early-stage anthocyanin synthesis enzyme CHI, which in turn impacts downstream DFR activity. This is consistent with the findings of Zhou et al. [24], where *CmCHS* expression was suppressed, leading to reduced expression of *CmDFR* and *CmANS* and a consequent decrease in anthocyanin synthesis. The high-temperature-sensitive chrysanthemum varieties, ‘Zi Hong Tuo Gui’ and ‘Zi Lian’, exhibited a more pronounced decrease in DFR enzyme activity following high-temperature stress, akin to the findings of Gal et al. [28], where a three-day high-temperature treatment significantly lowered anthocyanin synthesis in rose petals. In our study, ANS enzyme activity did not exhibit a consistent trend under the control, but ‘Chrystal Regal’ and ‘Zi Hong Tuo Gui’ showed a decreasing trend in ANS enzyme activity from stages S1 to S3 under high-temperature stress. Furthermore, after high-temperature treatment, ‘Zi Feng Che’ during stages S1 and S3, ‘Chrystal Regal’ during stages S1–S3, ‘Zi Hong Tuo Gui’ from stages S2 to S4, and ‘Zi Lian’ during stage S1 exhibited a significant decrease in ANS enzyme activity, mirroring the changes in anthocyanin content and underscoring the pivotal role of ANS enzyme activity in the reduction of anthocyanin content in petals under high-temperature stress.

Anthocyanin accumulation in petals is a complex biological process, influenced by environmental factors, enzyme activities, and gene expression, and involves synthesis, transport, and degradation, among other factors [29,30,31,32]. In our study, ‘Zi Lian’ demonstrated a marked increase in POD enzyme activity in petals across all developmental stages under high-temperature stress, with significantly higher POD enzyme activity than other varieties, which may be closely linked to the substantial reduction in anthocyanin content due to high temperatures. However, ‘Zi Feng Che’ and ‘Chrystal Regal’ also showed an increase in POD enzyme activity during stages S1 and S2 under high-temperature stress, but their anthocyanin content and petal coloration remained relatively stable. This could be attributed to a lower presence of phenolic substances that assist in POD enzyme function, resulting in a less pronounced degradation of anthocyanins by the POD enzyme. Additionally, ‘Zi Lian’ and ‘Zi Hong Tuo Gui’ exhibited a negative correlation between TAC and PPO enzymes’ activity under high-temperature stress, suggesting that the fading observed in these varieties may result from the combined action of PPO and POD enzymes, in line with previous studies indicating that PPO, POD, and βG are the main anthocyanin degradation enzymes [16,21,33,34]. Conversely, ‘Zi Feng Che’ displayed a significant decrease in βG enzyme activity across all developmental stages after high-temperature stress, which may explain the relative stability of anthocyanin accumulation in this variety’s petals under high temperatures. Furthermore, gene expression analyses during the S3 phase indicated that the Cse_sc027584.1_g010.1 (*PPO*) and Cse_sc031727.1_g010.1 (*POD*) genes might facilitate anthocyanin degradation in ‘Zi Hong Tuo Gui’ and ‘Zi Lian’ through upregulated expression under elevated temperatures, while maintaining expression equilibrium to conserve anthocyanin homeostasis in ‘Zi Feng Che’ and ‘Chrystal Regal’. Considering the vast array of genes implicated in anthocyanin biosynthesis and catabolism, pinpointing pivotal genes and unraveling the associated molecular mechanisms is challenging via qRT-PCR alone. Future endeavors will encompass multi-omics strategies, including metabolomics, transcriptomics, and proteomics, to comprehensively explore the molecular underpinnings influencing anthocyanin metabolism in chrysanthemums under thermal stress.

## 4. Materials and Methods

### 4.1. Plant Materials

This study utilized two high-temperature-sensitive chrysanthemum varieties, ‘Zi Hong Tuo Gui’ and ‘Zi Lian’, along with two high-temperature-stable varieties, ‘Zi Feng Che’ and ‘Chrystal Regal’, as experimental subjects (Figure 1). The vegetative growth phase of these chrysanthemum occurred at the Baiyun base of Guangzhou Houde Agricultural Science and Technology Co., Ltd. (Guangzhou, China), a resource nursery for chrysanthemum cultivation (113.23° E, 23.16° N). Cuttings were taken in September 2021, and by December, when the chrysanthemums displayed color and bloomed, specimens representing different flower developmental stages were selected for treatment in an intelligent light incubator. The conditions were set to high temperature (day 35 °C, 10 h, 20,000 Lx; night 26 °C, 14 h, 0 Lx) and normal temperature (day 24 °C, 10 h, 20,000 Lx; night 15 °C, 14 h, 0 Lx), with humidity maintained at 60%. Six pots per variety were marked and subjected to these conditions for seven days, with watering every two days. Post-treatment, flowers at stages bud (S1), early bloom (S2), early full bloom (S3), and mid–full bloom (S4) were harvested.

### 4.2. Determination of Total Anthocyanin, Flavonoid, and Phenolic Contents

The collected samples were ground into powders under liquid nitrogen using a multi-sample tissue grinder, then transferred to 2 mL cryogenic tubes and stored in a −80 °C freezer for later use. Weighing 0.1 g of frozen powder, it was placed into a 15 mL centrifuge tube, to which 5 mL of a 1% hydrochloric acid methanol extraction solution (concentrated hydrochloric acid:methanol = 1:99 (*v*/*v*)) was added. The mixture was shaken evenly and placed in a dark 4 °C environment for 24 h. It was then centrifuged at 4 °C, 10,000× *g*, for 15 min, and the supernatant was collected as the test solution for the determination of total anthocyanin, flavonoid, and phenolic contents.

The determination of total anthocyanin content followed the method of Liu et al. [35]. The absorbance of each sample’s test solution at 510 nm and 700 nm was measured using a UV-1200 spectrophotometer (MAPADA, Shanghai, China), with each sample being measured three times. The relative anthocyanin content (OD/g·FW) was calculated using the formula: ΔOD = (OD510,1−OD700,1) − (OD510,5−OD700,5). Here, OD510,1 represents the absorbance value at 510 nm under a pH of 1.0. The remaining terms follow a similar pattern.

The total flavonoid content was quantified using the methodologies outlined by Seeram et al. [36] and Li et al. [37]. A 10 mL test tube was prepared with 0.5 mL of the test solution, 2 mL of distilled water, and 0.15 mL of a 5% NaNO_2_ solution. This mixture was shaken until thoroughly mixed and then allowed to react for 6 min. Subsequently, a 10% Al(NO_3_)_3_ solution was introduced, mixed, and left to react for an additional 6 min. Finally, 2 mL of a 4% NaOH solution was added, and the total volume was adjusted to 5 mL with distilled water. After a 15 min reaction period at room temperature in the dark environment, the absorbance of each reaction solution was measured at 510 nm using a UV-1200 spectrophotometer (MAPADA, Shanghai, China). This process was repeated three times for each sample. The total flavonoid content was then calculated based on the standard curve and expressed as milligrams of rutin equivalent per gram of petal weight (mgRE/g FW). A standard curve was also established using a rutin standard solution and measured at 510 nm.

The total phenolic content was quantified using the Folin–Ciocalteu method [38]. A 10 mL test tube was prepared with 0.2 mL of the test solution, 4 mL of distilled water, and 0.5 mL of Folin–Ciocalteu reagent, added in that order. After a 5 min reaction period, 7% NaCO_3_ was added, and the total volume was adjusted to 10 mL with distilled water. The mixture was then left to react for 2 h at room temperature in a dark environment. The absorbance of each reaction solution was measured at 765 nm using a spectrophotometer. This process was repeated three times for each sample. The total phenolic content was then calculated based on the standard curve and expressed as milligrams of gallic acid equivalent per gram of petal weight (mgGAE/g FW). A standard curve for total phenolic content was also established using gallic acid standard solution and measured at 765 nm.

### 4.3. Determination of Enzyme Activity

Each sample of frozen powder, weighing 0.05 g, was placed into a pre-cooled, clean, 2 mL centrifuge tube. Subsequently, 1.5 mL of PBS phosphate buffer (pH 7.0, 0.05 M/L) was swiftly added. The mixture was vortexed for one minute to create a homogenate, then centrifuged at 4 °C, 12,000 rpm, for 20 min. The supernatant was transferred to a new clean centrifuge tube and stored at 4 °C, to be tested within three days.

The activity of the POD enzyme was determined with minor modifications to the method proposed by Wang et al. [39]. The POD assay system, with a total volume of 3 mL, consisted of 2.65 mL of 0.05 M phosphate buffer (pH 7.0), 0.2 mL of 0.2 M guaiacol, and 0.1 mL of 0.15 M H_2_O_2_. The reaction was initiated by adding 0.05 mL of the enzyme solution, and the change in OD470 was measured using a UV-Vis spectrophotometer in kinetic mode over a period of two minutes. The activity was calculated thrice using the formula: enzyme activity (U/(h·mL)) = (OD470_final_ − OD470_initial_) × 30/0.01/0.05.

The enzyme activities of PAL, CHI, DFR, ANS, PPO, and β-glucosidase (βG) were detected using ELISA kits [40].

### 4.4. Quantitative Real-Time PCR (qRT-PCR) Analysis

Synthesis of single-stranded cDNA from sequenced RNA samples was performed using the HiScript II Q RT SuperMix for qPCR kit (Vazyme, Nanjing, China), following the manufacturer’s protocol. An initial volume of 1 µg of RNA was mixed with RNase-free ddH2O to reach 8 µL in an RNase-free PCR tube. This mixture was incubated at 65 °C for 5 min, then rapidly cooled on ice for 2 min. Next, 2 µL of 5 × gDNA wiper Mix was added, the tube was flicked for mixing, and it was incubated at 42 °C for 2 min. Following the preparation of the reaction mixture, the reverse transcription was executed with a program set at 25 °C for 5 min, 37 °C for 45 min, and 85 °C for 5 s. The resultant cDNA was stored at −20 °C. The 2X SYBR Green Abstart PCR Mix (Sangon Biotech, Shanghai, China) served as the premix. The four genes selected for quantitative real-time PCR (qRT-PCR) analysis—Cse_sc021211.1_g010.1 (*CHI*), Cse_sc003501.1_g030.1 (*ANS*), Cse_sc027584.1_g010.1 (*PPO*), and Cse_sc031727.1_g010.1 (*POD*)—were sourced from the *Chrysanthemum seticuspe* genomic database [41]. Primers, designed via the Primer 3 website (https://primer3.ut.ee/, accessed on 20 May 2024) with *CmEF1α* as the internal control, are listed in Table S5 [42]. Gene expression was quantified using the 2^−ΔΔCT^ method.

### 4.5. Data Statistical Analysis

All index assays were performed with three biological replicates. One-way analysis of variance and Pearson correlation analysis were performed using built-in functions in R language [43]. OPLS-DA was performed using the “MetaboAnalystR” package, and VIP values for variables were calculated [44]. The “corrplot” package was utilized for heatmap visualization analysis [45].

## 5. Conclusions

The study found that ‘Zi Feng Che’ and ‘Chrystal Regal’ maintained their color and anthocyanin glycoside levels under high-temperature stress. In contrast, ‘Zi Hong Tuo Gui’ and ‘Zi Lian’ experienced significant color fading and anthocyanin reduction during the early bloom stages. The enzymatic activities of CHI, DFR, and ANS were closely associated with these changes, particularly during critical developmental stages. ‘Zi Lian’ exhibited a unique response with an increase in POD activity, suggesting a complex interplay between enzymes, affecting color stability. The decrease in βG activity in ‘Zi Feng Che’ indicated its role in maintaining color consistency. The genes Cse_sc027584.1_g010.1 (*PPO*) and Cse_sc031727.1_g010.1 (*POD*) may promote anthocyanin degradation in ‘Zi Hong Tuo Gui’ and ‘Zi Lian’ via increased expression under high temperatures, while preserving expression balance to maintain anthocyanin homeostasis in ‘Zi Feng Che’ and ‘Chrystal Regal’ at the early bloom floral stage. In summary, this research enhanced our understanding of the thermal response of chrysanthemum pigmentation and enzymatic behavior.

## Data Availability

Data are contained within the article and Supplementary Materials.

## Supplementary Materials

The following supporting information can be downloaded at: https://www.mdpi.com/article/10.3390/plants13131865/s1. Table S1: Pearson correlation coefficient among ten physiological indicators for ‘Zi Feng Che’. Table S2: Pearson correlation coefficient among ten physiological indicators for ‘Chrystal Regal’. Table S3: Pearson correlation coefficient among ten physiological indicators for ‘Zi Hong Tuo Gui’. Table S4: Pearson correlation coefficient among ten physiological indicators for ‘Zi Lian’. Table S5: Primer sequences for qRT-PCR analysis.
